# Directly targeting G-quadruplexes contributes to the anti-multiple myeloma efficacy of Epimedokoreanin B: Identification of EKB as a potential G4 stabilizer

**DOI:** 10.3724/abbs.2025110

**Published:** 2025-08-14

**Authors:** Pingting Jia, Shangzhao Wang, Wanting Huang, Ye Fang, Jian Gao

**Affiliations:** 1 School of Medicine Anhui University of Science and Technology Huainan 232001 China; 2 The First Affiliated Hospital of Anhui University of Science and Technology (Huainan First People’s Hospital) Huainan 232007 China

**Keywords:** multiple myeloma, G-quadruplexes, Epimedokoreanin B, fluorescence resonance energy transfer, molecular dynamics

## Abstract

Multiple myeloma (MM) is a hematological malignancy for which novel therapeutic strategies are urgently needed. Epimedokoreanin B (EKB), an isoprenylated flavonoid compound derived from the medicinal plant *Epimedium koreanum*, has demonstrated promising antitumor activity. However, its effects on MM have not been previously investigated. This study explores the anti-MM activity and the molecular interaction mechanisms between EKB and G-quadruplexes (G4) through a combination of biological activity assessments and computer-aided methodologies. EKB exhibits potent cytotoxicity against the MM cell lines U266 and RPMI-8226, with IC _50_ values of 5.28 μM and 6.81 μM, respectively. It induces apoptosis in a concentration-dependent manner and specifically stabilizes the G4 structures of oncogenes such as *c-Myc*, *c-KIT*, *Bcl-2*, and *k-RAS*, as confirmed by BG4 immunofluorescence staining and fluorescence resonance energy transfer (FRET) assays. Additionally, EKB significantly suppresses the mRNA and protein expression levels of these genes in myeloma cells. Computational studies, including molecular docking, molecular dynamics (MD) simulations, and MM/GBSA calculations, confirm the strong binding affinity and stabilizing effects of EKB on G4s, revealing a mechanism involving π-π stacking and hydrogen bonding. This discovery underscores the unique ability of EKB to increase the stability of G4 structures, which are critical for regulating gene expression and inhibiting cancer cell proliferation. This research highlights the therapeutic potential of EKB in targeting these specific molecular structures, thereby offering a more effective approach to managing MM.

## Introduction

Multiple myeloma (MM) is a hematologic malignancy characterized by the abnormal proliferation of plasma cells within the bone marrow and excessive production of monoclonal immunoglobulin or light chains (M protein). It is the second most common hematologic malignancy [1]. MM predominantly affects elderly individuals, with a higher incidence in males than in females. As the population ages, the incidence of MM is expected to increase annually [2]. Despite significant advancements in treatment modalities, including proteasome inhibitors, immunomodulatory drugs, and monoclonal antibodies, MM remains incurable, with drug resistance posing a major challenge. Furthermore, relapsed/refractory multiple myeloma (RRMM) has emerged as a critical focus, necessitating the development of novel therapeutic agents and strategies [ 3, 4].

**Figure 1:**
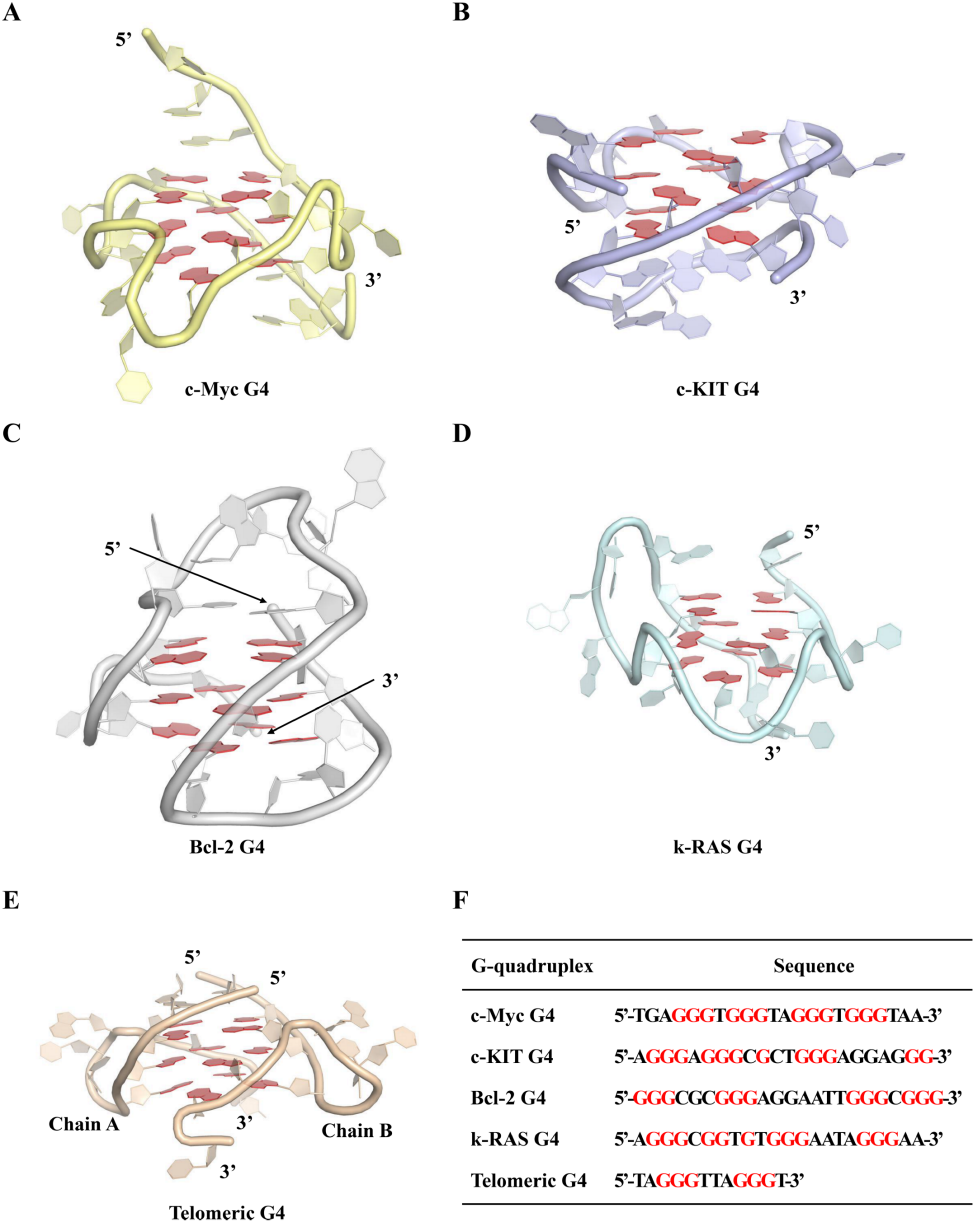
Figure 1 Structures and sequences of G4 DNA motifs (A–E) Crystal structures of the c-Myc G4, c-KIT G4, Bcl-2 G4, k-RAS G4, and telomeric G4 DNA motifs. (F) Nucleotide sequences of the five aforementioned G4 structures.

Aberrant activation of the oncogene c-Myc plays a critical role in various types of cancer, including serous ovarian cancer, breast cancer, lung cancer, leukemia, and multiple myeloma [ 5– 7]. Furthermore, the nuclease hypersensitive element III1 (NHE III1) region upstream of the P1 promoter of the *c-Myc* gene, which contains a purine-rich DNA sequence, can form a specialized secondary structure known as a G-quadruplex (G4) [8]. This structure is well known for its role as a transcriptional repressor [8]. Previous studies have demonstrated that the stabilization of these structures through interactions with small molecules could lead to the downregulation of oncogene expression [5]. Therefore, the G4 structure of the cancer-related gene *c-Myc* has long been considered a potential drug target [9]. Subsequently, numerous G4 structures have been identified in the promoters of various oncogenes, including *c-KIT*, *Bcl-2*, and *k-RAS* ( Figure 1). These discoveries have since positioned G4s as highly attractive targets for the development of antitumor therapeutics [ 10– 13] .

G4 structures are widely distributed in the promoter regions of various oncogenes and play crucial regulatory roles in tumor initiation and progression [14]. As such, they have emerged as highly promising targets for anticancer therapy. With increasing research, it has become evident that G4 structures are formed by the folding of single-stranded DNA into highly ordered conformations, exhibiting spatial specificity akin to the binding pockets found in globular proteins. This structural feature provides a precise platform for small-molecule binding, enabling targeted stabilization of G4 structures to achieve transcriptional repression of oncogenes [15]. Accordingly, small-molecule targeting of G4s has become a key strategy in anticancer drug discovery [16]. This approach not only addresses the limitations of sequence specificity encountered with conventional linear DNA targets but also offers a novel avenue for the development of mechanism-based therapeutics [17]. In recent years, significant progress has been made in the identification of natural products capable of selectively targeting DNA G4 structures. Several G4 motifs—such as c-Myc G4, k-RAS G4, PDGFR-β G4, Bcl-2 G4, VEGF G4, and telomeric G4—have been validated as effective binding targets for natural small molecules [18], thereby laying a solid foundation for the development of G4-targeted anticancer agents derived from natural products. Notably, approximately 64.9% of approved anticancer drugs are either directly or indirectly derived from natural products, including well-known agents such as paclitaxel, camptothecin, doxorubicin, and etoposide [18]. Owing to their structural diversity and potent biological activity, natural products continue to serve as invaluable reservoirs for the discovery of novel G4 stabilizers with therapeutic potential.

**Figure 2:**
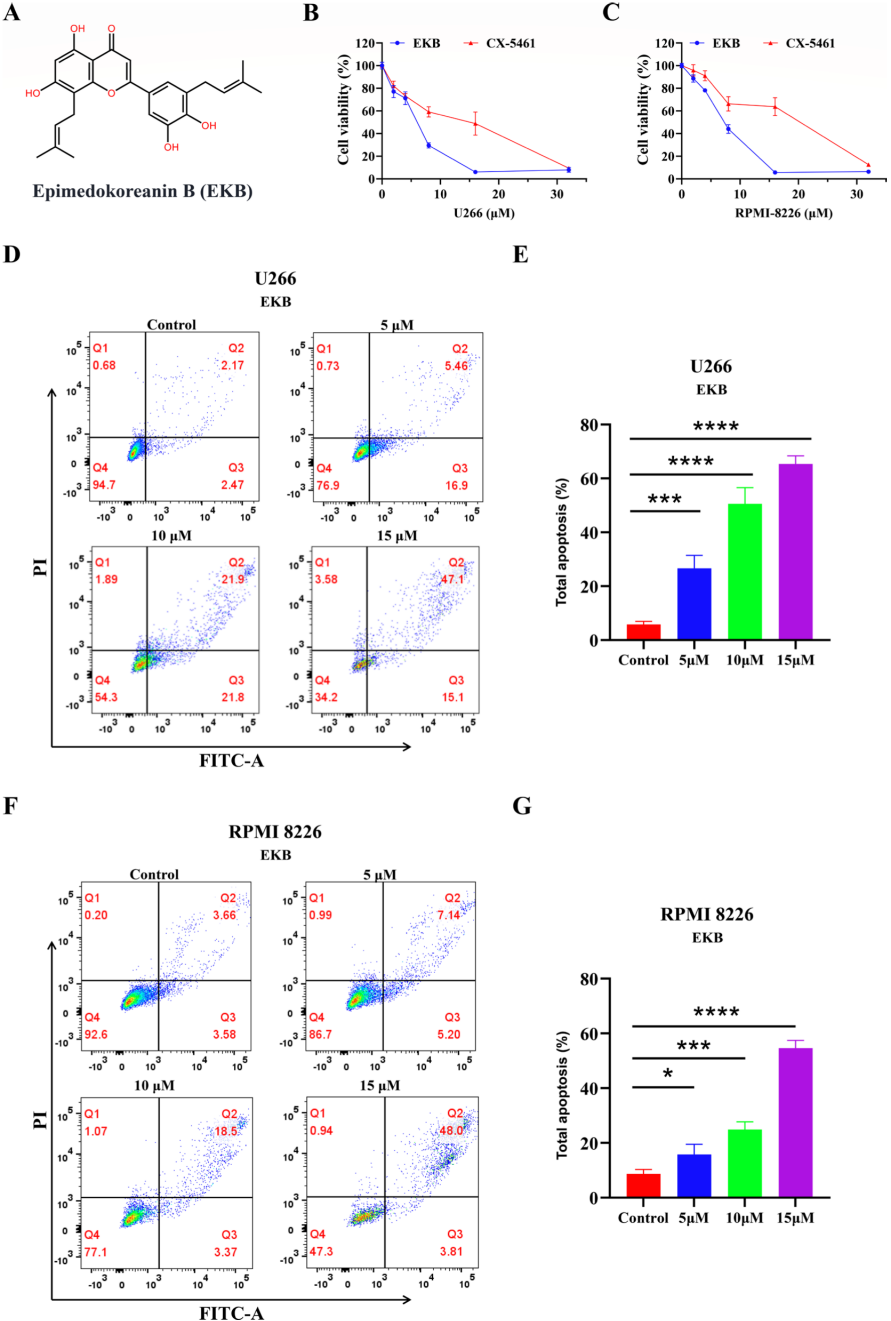
Figure 2 Anti-MM activity of EKB in U266 and RPMI-8226 cells (A) Chemical structure of EKB. (B,C) Effects of different concentrations of EKB and CX-5461 on the viability of U266 and RPMI-8226 cells. EKB induced apoptosis in U266 (D,E) and RPMI-8226 (F,G) cells. Both cell lines were treated with EKB at concentrations of 5, 10, and 15 μM for 48 h. Data are shown as the mean ± SD (n = 3). Statistical significance was assessed by one-way ANOVA with Tukey’s test (*P < 0.05, ***P < 0.001, ****P < 0.0001).

Epimedokoreanin B (EKB) is an isoprenylated flavonoid compound isolated from the medicinal plant *Epimedium koreanum* ( Figure 2A) [19]. EKB exhibits a wide range of pharmacological activities, with particularly notable effects in antitumor applications. In 2019, Kariu *et al*. [20] reported that EKB effectively inhibited toxicity in culture supernatants containing subgingival plaque bacteria, suggesting its potential as a novel therapeutic agent against periodontal pathogens with promising application prospects. In 2020, Pan *et al*. [21] demonstrated that EKB suppressed tumor progression by inhibiting STAT3 activation within the tumor microenvironment. More recently, in 2022, Zheng *et al*. [19] revealed that EKB inhibited lung cancer cell growth via endoplasmic reticulum stress-mediated paraptosis, which was characterized by the accumulation of autophagosomes. Furthermore, EKB inhibited lung cancer cell proliferation in a zebrafish xenograft model [19]. To date, no studies have been published on the inhibition of myeloma cells by EKB through the targeting of G4 structures. The specific targeting ability and selectivity of this compound toward G4 warrant further investigation.

In the present study, we systematically evaluated the cytotoxicity, apoptotic effects, and changes in protein expression levels induced by EKB using CCK-8 assay, apoptosis analysis, and western blot analysis. To further assess the G4-targeting specificity and selectivity of EKB, BG4 immunofluorescence staining, fluorescence resonance energy transfer (FRET) assay, and quantitative real-time PCR (q-PCR) were conducted. Additionally, molecular docking was utilized to analyze the binding conformations of EKB with G4 structures, whereas molecular dynamics (MD) simulations were conducted to investigate the stability and dynamic behavior of EKB-G4 interactions.

## Materials and Methods

### Cell culture

The human myeloma cell lines RPMI-8226 and U266 (Shanghai Institute of Biochemistry and Cell Biology, Chinese Academy of Sciences, Shanghai, China) were maintained in RPMI-1640 medium (KeyGEN BioTECH, Shanghai, China) supplemented with 10% fetal bovine serum (FBS). The cells were cultured in a humidified incubator at 37°C with 5% CO _2_ for 48 h.

### Cell viability assay

RPMI-8226 and U266 cells were seeded in 96-well plates at a density of 5000 cells per well. The cells were treated with various concentrations of the test compounds (2, 4, 8, 16, and 32 μM), while the cells treated with DMSO alone served as the vehicle control. The plates were incubated for 48 h in a humidified atmosphere containing 5% CO _2_ at 37°C. Following the treatment period, 10 μL of CCK-8 solution (TargetMol, Shanghai, China) was added to each well, and the plates were further incubated under the same conditions for an additional 3 h. Cell viability was assessed by measuring the absorbance at 450 nm using a microplate reader (BioTek, Winooski, USA), and the data were analyzed to determine cell viability.

### Cell apoptosis

Annexin V conjugated with fluorescein isothiocyanate (FITC) (Servicebio, Wuhan, China) was used as a fluorescent probe to bind to phosphatidylserine (PS) on the outer leaflet of the cell membrane, thereby indicating membrane integrity. Propidium iodide (PI), a nucleic acid dye, stains nuclei red. The combination of these two reagents allows for the precise determination of different stages of apoptosis. RPMI-8226 and U266 cells were seeded in 12-well plates at a density of 15,000 cells per well (approximately 50%–60% confluence). Following 48 h of drug treatment, the cells were harvested and washed with PBS. The cells were then resuspended in 100 μL of binding buffer, and 1 μL each of FITC and PI was added to samples treated with various concentrations of the drug. The mixture was incubated at room temperature for 20–30 min. Subsequently, the number of apoptotic cells was quantitatively analyzed using a flow cytometer (BD Biosciences, Franklin Lakes, USA).

### Quantitative PCR

The cells were lysed with 0.5 mL of Trizol reagent (ABclonal, Wuhan, China) through vigorous shaking and incubated at room temperature for 3–5 min. Subsequently, 200 μL of chloroform was added, followed by vigorous shaking and centrifugation at 10,000 *g* for 15 min. The supernatant was collected and subjected to isopropyl alcohol precipitation, after which it was centrifuged for approximately 30 min. The pellet was washed twice with 70% ethanol. The RNA was then air-dried and resuspended in RNase-free water to assess its purity and concentration. Following purification, cDNA was synthesized by reverse transcription and analyzed using the QuantStudio3 real-time PCR system (Thermo Fisher Scientific, Waltham, USA). Data from amplification curves of various drug concentrations were processed and compared against the control group to evaluate the effects of the drugs on cellular activity.

### Western blot analysis

The cells were lysed with RIPA buffer (Beyotime, Shanghai, China). The resulting protein lysates were centrifuged to collect the supernatant. Protein concentrations were determined using the BCA reagent (Servicebio), and samples were prepared by heating at 100°C for 5 min. Electrophoresis was performed to separate the proteins, which were subsequently transferred onto PVDF membranes (Millipore, Billerica, USA) via wet electroblotting. The membranes were blocked with 5% non-fat milk in TBST for 1–2 h, followed by three washes with TBST (5 min each). Primary antibodies used in this study included GAPDH rabbit mAb (A19056; ABclonal), c-Myc rabbit mAb (A19032; ABclonal), c-Kit rabbit pAb (A7521; ABclonal), Bcl-2 rabbit mAb (A19693; ABclonal), and KRAS rabbit mAb (A23382; ABclonal). HRP-conjugated goat anti-rabbit IgG (H+L) (AS014; ABclonal) was used as the secondary antibody. All primary antibodies were incubated overnight at 4°C, followed by incubation with the secondary antibody for 1 h at room temperature. Protein expression was detected using the ECL method to evaluate the effect of the drug on the cells.

### Immunofluorescence staining

The cells were fixed with 4% paraformaldehyde (Beyotime) at room temperature for 20 min, transferred to poly-L-lysine-coated slides, air-dried, and washed with PBS. Permeabilization was carried out using 0.2% Triton X-100 (Beyotime) for 10 min at room temperature, followed by additional PBS washes. Nonspecific binding was blocked with 1% BSA for 1 h at room temperature. The cells were then incubated overnight at 4°C with the BG4 primary antibody (MedChemExpress, Monmouth Junction, USA), washed with PBS, and treated with a FITC-conjugated secondary antibody (Servicebio) under dark conditions for 1 h. After washing with PBS, the nuclei were stained with DAPI (Servicebio) for 10 min at room temperature. The slides were mounted and imaged using an inverted fluorescence microscope (DMi8; Leica, Wetzlar, Germany) to analyze the fluorescence signal distribution of the G4 structures.

### Fluorescence resonance energy transfer

Fluorescence resonance energy transfer (FRET) was used to determine whether target genes interact with G4 structures. This experiment focused primarily on five targets, including double-stranded DNA (dsDNA), and the G4 structures associated with the c-Myc, c-KIT, k-RAS, Bcl-2, and telomeric regions. G4-structured oligonucleotides corresponding to these gene sequences, along with a dsDNA control ( Supplementary Table S1), were synthesized and fluorescently labelled (Sangon Biotech, Shanghai, China). For the fluorescence assays, 2 μL of each oligonucleotide was added to a 200 μL Tris-HCl buffer system containing 50 mM KCl (pH 7.4), mixed thoroughly, and centrifuged briefly to remove bubbles. The oligonucleotides were subsequently heated to 95°C for 5 min, cooled to room temperature, and incubated at 4°C for 24 h in the dark. FRET detection was conducted using the real-time PCR detection system (QuantStudio3; Thermo Fisher Scientific) to monitor the fluorescence determination of the melting temperature (Tm) of the G4 structure in the presence of EKB. Each experiment was independently repeated at least three times ( *n* ≥ 3).

### Dual-luciferase reporter assay

293T cells were transfected with a plasmid containing the wild-type c-Myc promoter (DEL64, Addgene plasmid #16604; Addgene, Cambridge, USA) [22] using Lipofectamine 2000 (Thermo Fisher Scientific). After 24 h, the cells were treated with EKB at concentrations of 5, 10, and 15 μM, followed by an additional 24-h incubation. Luciferase activity was measured using the Dual-Luciferase Reporter Assay System (Promega, Madison, USA), with Renilla luciferase activity normalized to firefly luciferase activity.

### Molecular docking

In this study, we selected the crystal structures of G4 motifs from c-Myc G4 (PDB ID: 2L7V [23]), c-KIT G4 (PDB ID: 4WO2 [24]), Bcl-2 G4 (PDB ID: 2F8U [25]) and k-RAS G4 (PDB ID: 7X8O [26]) as targets to investigate the potential binding of EKB. Our objective was to elucidate the binding mechanism and specific interaction patterns between EKB and these G4 structures. Prior to analysis, the four DNA crystal structures were preprocessed by removing water molecules, hydrogen atoms, and other small molecules to ensure the structural integrity of the DNA and accurate charge distribution for subsequent pairing studies. The Surflex-Dock module within Sybyl-X 2.1 software was utilized for this study, in which we constructed receptor pocket structures for molecular docking. The ligand EKB was preprocessed following standard protocols and subsequently docked into the generated pocket. The alignment structures with higher scores were selected for subsequent molecular dynamics simulations. To improve docking performance, we increased the number of initial ligand poses, expanded the sampling space, incorporated flexible ring conformations, and optimized the ligand structures prior to docking.

### Molecular dynamics

The docked conformations of EKB bound to G4s served as the initial structures for the MD simulations. Antechamber was utilized to process small molecules, assigning RESP atomic charges and GAFF force field parameters to generate the corresponding topology and force field files. The complex was positioned at the center of a cubic water box composed of TIP3P water molecules. For G4s, we employed the ff99bsc1 force field, whereas GAFF was used for small-molecule compounds to establish the necessary parameters for MD simulations. System energy minimization was conducted using the sander module in Amber18. The system was gradually heated from 0 K to 300 K within 60 ps. A 200 ns MD simulation was performed at a constant temperature of 300 K with a time step of 2 fs. Root mean square deviation (RMSD) and root mean square fluctuation (RMSF) analyses were conducted to assess the dynamic stability of each system, and the binding free energy between the ligand EKB and the target G4 was calculated using the MM/GBSA method.

### UV-visible absorption titration assay

UV-visible absorption titration experiments were conducted to evaluate the binding interactions between EKB and G4 DNA structures (Sangon Biotech). All the experiments were performed in 10 mM Tris-HCl buffer containing 100 mM KCl (pH 7.4) to mimic the physiological ionic conditions favourable for G4 formation. The concentration of EKB was fixed at 20 μM, and G4 oligonucleotides derived from the promoter regions of c-Myc, Bcl-2, c-KIT, and k-RAS were gradually added at increasing molar ratios (G4:EKB = 1:1 to 8:1).

### Statistical analysis

Statistical analyses were conducted using GraphPad Prism 10 (GraphPad Software, La Jolla, USA). Data are expressed as the mean ± standard deviation. Intergroup comparisons were evaluated via two-way analysis of variance (ANOVA). A significance threshold of *P* < 0.05 was applied for all the statistical assessments.

## Results

### EKB inhibits MM cell viability and induces apoptosis

To investigate the anti-MM activity of EKB, we selected two established MM cell lines, U266 and RPMI-8226. The cytotoxic effects of EKB on these cell lines were assessed using the CCK-8 assay. The IC _50_ values of EKB in U266 and RPMI-8226 cells were determined to be 5.28 μM and 6.81 μM, respectively, for comparison, the IC _50_ values of the known G4 stabilizer CX-5461 [27] were 14.12 μM in U266 cells and 18.54 μM in RPMI-8226 cells, suggesting that EKB is more cytotoxic than CX-5461 in both cell lines ( Figure 2B,C). These results indicate that EKB exerts significant inhibitory effects on both cell lines. Therefore, EKB might represent a promising candidate for further investigation as an anticancer agent.

To further evaluate the anti-MM activity of EKB, we conducted apoptosis studies on U266 and RPMI-8226 cells. As shown in Figure 2D–G, EKB exhibited concentration-dependent apoptotic effects in both cell lines. Specifically, at a concentration of 5 μM, EKB induced an apoptosis rate of 22.36% in U266 cells. This percentage increased to 62.2% at 15 μM. Similarly, for the RPMI-8226 cells, the percentage of apoptotic cells increased from 12.34% at 5 μM to 51.81% at 15 μM. These findings demonstrate that EKB significantly promotes apoptosis in both U266 and RPMI-8226 cells at concentrations of 5 μM, 10 μM, and 15 μM in a concentration-dependent manner.

### Stability analysis of EKB on G4s

**Figure 3:**
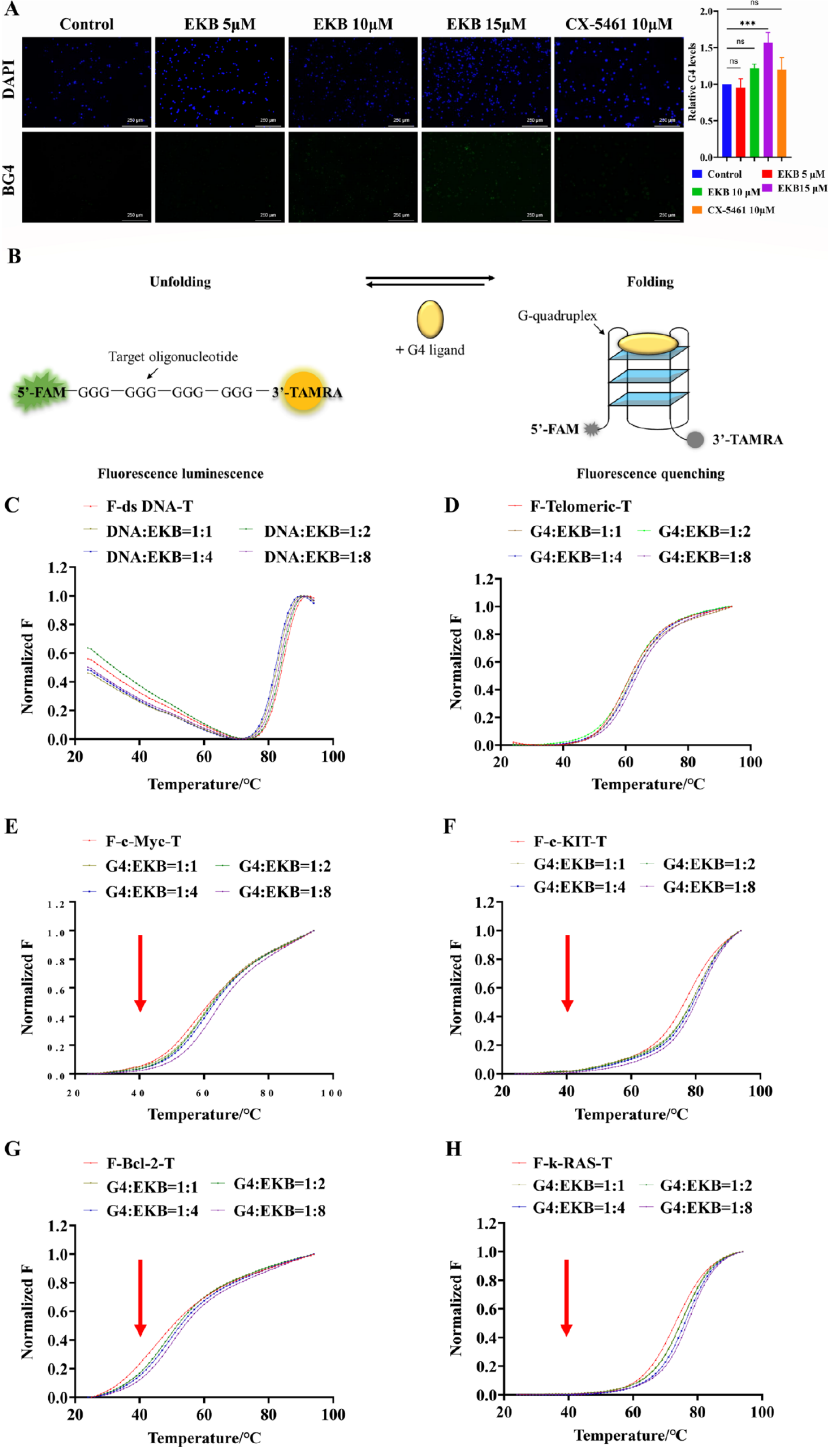
Figure 3 EKB enhances G4 formation in cells and stabilizes G4 structures *in vitro* (A) BG4 immunofluorescence staining of RPMI-8226 cells treated with EKB. RPMI-8226 cells were treated with EKB (5, 10, or 15 μM) or CX-5461 (10 μM) for 48 h. G4s were detected via the BG4 antibody (green), and the nuclei were stained with DAPI (blue). The mean fluorescence intensity over the entire image was quantified via ImageJ (mean = IntDen/area). (B) The principle of FRET. (C–H) Stabilization effects of EKB on dsDNA and G4s at various concentrations. Oligonucleotides were used at a concentration of 1 μM, and EKB was added at 0, 1, 2, 4, and 8 molar equivalents relative to the oligonucleotides. Data are shown as the mean ± SD (n = 3). Statistical significance was assessed by one-way ANOVA with Tukey’s test (ns, not significant, ***P < 0.001).

We subsequently validated the G4-stabilizing effects of EKB and CX-5461 in a cellular context. Immunofluorescence staining was performed in RPMI-8226 cells using the G4-specific antibody BG4 [28]. As shown in Figure 3A, green fluorescence corresponding to BG4 staining was barely detectable in the control group, indicating minimal basal formation of G4 structures. Upon treatment with increasing concentrations of EKB, the intensity of BG4 foci increased in a dose-dependent manner. Notably, at 10 μM, EKB induced a marked increase in BG4 fluorescence comparable to that observed with the positive control CX-5461 (10 μM), suggesting that EKB has a similar capacity to stabilize G4 structures. At 15 μM, the fluorescence signal was further elevated, indicating increased accumulation of G4 structures.

To evaluate the stabilizing effects of EKB on various G4 DNA structures, FRET-based melting assays were conducted ( Figure 3B). Double-stranded DNA (dsDNA) was used as a negative control to assess the G4 selectivity of EKB. In the dsDNA group, the melting temperature (Tm) slightly decreased with increasing concentrations of EKB ( Table 1 and Figure 3C), but no significant or consistent trend was observed, suggesting a structural preference of EKB for G4 over canonical duplex DNA. For the telomeric G4 oligonucleotide, the Tm increased modestly from 61.44°C (negative control) to 63.56°C at an 1:8 G4:EKB molar ratio ( Supplementary Table S2), with ΔTm values ranging from 0.12°C to 2.12°C. However, this change did not exhibit a concentration-dependent pattern, indicating a limited stabilizing effect of EKB on telomeric G4s ( Figure 3D). This result may be attributed to the structural polymorphism of telomeric G4s, including variations in G-tetrad stacking and loop arrangements [17], which could contribute to reduced binding specificity.

**Table:** **Table 1** Thermal stabilization of EKB in response to diverse target oligonucleotides

*ΔT _m_*/°C	dsDNA	Telomeric	c-Myc	c-KIT	Bcl-2	k-RAS
*ΔT _m_* (1 eq)	–1.87	0.68	0.78	3.56	4.41	1.11
*ΔT _m_* ​ (2 eq)	–0.40	0.12	1.11	3.11	4.39	1.00
*ΔT _m_* (4 eq)	–2.30	1.15	1.81	3.69	5.53	1.68
*ΔT _m_* ​ (8 eq)	–1.16	2.12	3.90	4.17	7.09	3.22

In contrast, EKB clearly demonstrated concentration-dependent stabilization of certain G4 structures. For the c-Myc G4 oligonucleotide, the Tm increased from 61.76°C to 65.66°C, with ΔTm values increasing from 0.78°C at a 1:1 molar ratio to 3.90°C at 1:8 ( Figure 3E). Similar stabilization was observed for c-KIT G4 (Tm from 78.33°C to 82.50°C) and Bcl-2 G4, which presented the largest ΔTm of 7.09°C (from 45.65°C to 52.74°C) at 8 equivalents ( Table 1 and Figure 3F,G). The k-RAS G4 also exhibited enhanced thermal stability, with the Tm increasing from 55.33°C to 58.55°C ( Figure 3H).

A UV-visible absorption assay was subsequently performed, revealing a redshift in the absorption peak of G4 DNA upon EKB treatment ( Supplementary Figure S1), further confirming the direct interaction between the small molecule and G4 DNA. Taken together, these data indicate that EKB is a G4-binding and G4-stabilizing small molecule capable of selectively stabilizing multiple G4 structures at both the molecular and the cellular level.

### The impact of EKB on the expression levels of oncogenes

**Figure 4:**
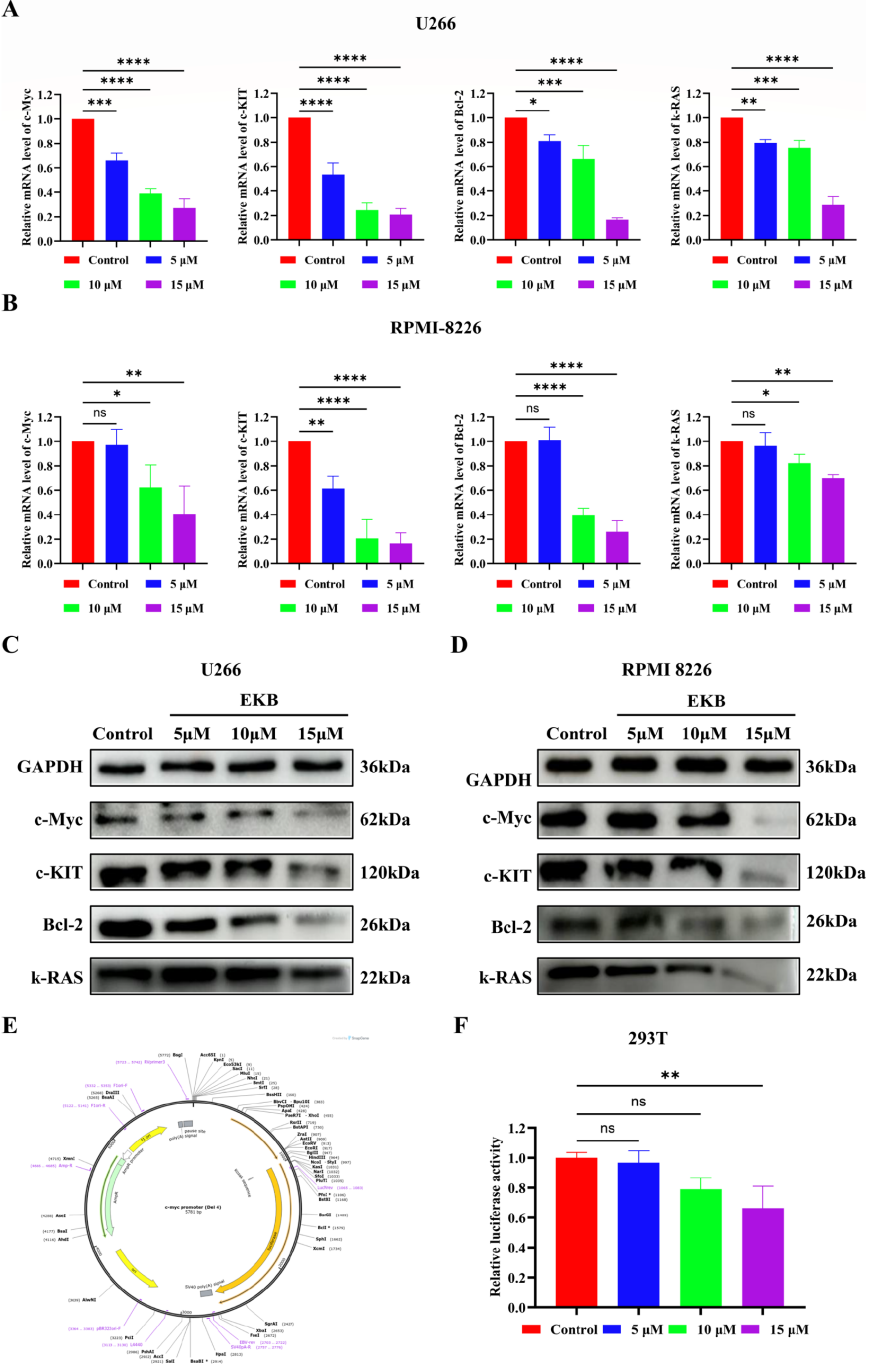
Figure 4 Effects of EKB on oncogene expression (A) The influence of EKB on the relative mRNA expression levels of c-Myc, c-KIT , Bcl-2 and k-RAS in U266 cells. (B) The influence of EKB on the relative mRNA expression levels of c-Myc , c-KIT, Bcl-2 and k-RAS in RPMI-8226 cells. (C,D) Influence of EKB on the expression levels of the c-Myc, c-KIT, Bcl-2 and k-RAS proteins in U266 and RPMI-8226 cells. (E) Schematic representation of the c-Myc promoter-luciferase reporter plasmid used for transcriptional activity analysis. (F) Relative luciferase activity in 293T cells transfected with the c-Myc promoter-luciferase construct and treated with EKB for 24 h. Data are shown as the mean ± SD ( n = 3). Statistical significance was assessed by one-way ANOVA with Tukey’s test (ns, not significant, *P < 0.05, **P < 0.01, ***P < 0.001, ****P < 0.0001).

The stabilization of G4s can lead to a significant reduction in the expression levels of oncogenes [ 18, 29 – 32]. Therefore, examining the impact of EKB on the expression levels of cancer-related genes, particularly *c-Myc*, *c-KIT*, *Bcl-2* and *k-RAS*, in the U266 and RPMI-8226 cell lines is crucial. q-PCR analysis revealed that EKB treatment led to a dose-dependent reduction in the mRNA expression levels of all four genes in both cell lines ( Figure 4A,B). Among these, *c-KIT* showed the most pronounced response, followed by *c-Myc*, *Bcl-2*, and *k-RAS* .

Moreover, western blot analysis revealed that EKB inhibited c-Myc, c-KIT, Bcl-2 and k-RAS expressions in both cell lines in a concentration-dependent manner ( Figure 4C,D). Notably, at a concentration of 5 μM, EKB had early-stage inhibitory effects on the expressions of these oncogenes, which became significantly more pronounced at 15 μM ( Supplementary Figure S2). Collectively, these findings suggest that EKB effectively suppresses the expressions of the c-Myc, c-KIT, Bcl-2 and k-RAS proteins, underscoring its potential as an antitumor therapeutic.

To investigate whether EKB can repress transcription by binding to and stabilizing G4 structures, we used the well-characterized c-Myc G4 promoter as a representative model and conducted a dual-luciferase reporter assay with a plasmid containing the *c-Myc* promoter region ( Figure 4E). This region includes NHE III1, which is known to form G4 structures critical for the transcriptional control of *c-Myc*. The assay revealed that EKB significantly reduced luciferase activity in a dose-dependent manner, particularly at 15 μM, indicating that EKB likely represses c-Myc expression by stabilizing G4 structures within this regulatory region ( Figure 4F).

These findings suggest that EKB suppresses oncogenic signaling by targeting G4-forming sequences in key cancer-related genes, especially within transcriptionally active, G4-enriched regulatory domains such as the c-Myc NHE region, underscoring its potential as a multitarget antitumor agent.

### MD simulation of G4/EKB complexes

**Figure 5:**
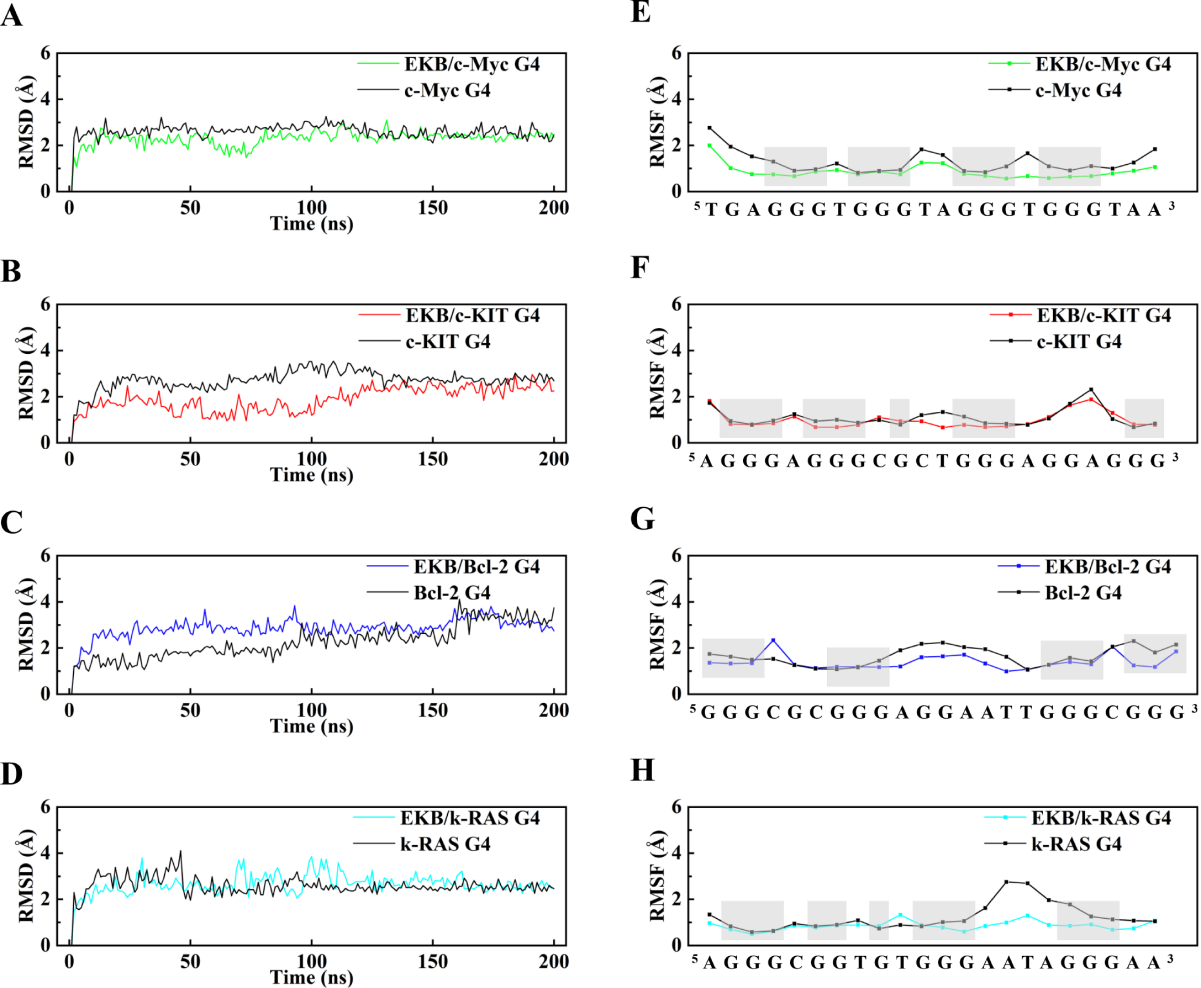
Figure 5 MD simulation of G4/EKB complexes (A–D) RMSD over a 200-ns period for the G4 structures and their respective complexes with EKB. (E–H) RMSF plots for the G4 structures and their respective complexes with EKB. The guanine residues that constitute the G4 structure are highlighted in gray.

To examine the stabilizing effects of EKB on various G4 structures, we analyzed the root mean square deviation (RMSD) values ( Figure 5A–D). The RMSD analysis revealed that all the complex systems gradually reached stability after 20 ns, with average RMSD values ranging from 1.9 Å to 2.9 Å. In contrast, the ligand-free G4 systems presented RMSD values between 2.3 Å and 2.7 Å. Notably, the c-Myc G4/EKB system achieved equilibrium most rapidly, indicating robust and efficient stabilization. Although the c-KIT G4/EKB system required additional time to stabilize, it demonstrated lower fluctuations postequilibrium. The Bcl-2 G4/EKB system rapidly stabilized in the initial phase, followed by a slight increase in the RMSD at 150 ns, which promptly returned to the mean value. Finally, the k-RAS G4/EKB system experienced initial fluctuations but exhibited enhanced stability in later stages.

RMSF analysis further elucidated the localized effects of EKB on G4 structures ( Figure 5E–H). The RMSF values for the c-Myc G4/EKB and Bcl-2 G4/EKB systems exhibited substantial reductions in base fluctuations, underscoring the ability of EKB to stabilize G4 structures. While the stabilizing effect on the c-KIT G4/EKB system was less pronounced, it remained evident. In the k-RAS G4/EKB system, the influence of EKB was more localized, with pronounced stabilization observed up to base position 13, whereas weaker effects were noted beyond this point, possibly due to spatial constraints. Collectively, these findings indicate that EKB effectively enhances the stability of G4-DNA structures. However, the extent and nature of its stabilizing effects vary across different systems, reflecting the dynamic structural characteristics and interaction patterns of each G4.

### MM/GBSA binding free energy calculation

The relative binding affinities of EKB with c-Myc G4, c-KIT G4, Bcl-2 G4, and K-RAS G4 were evaluated via the MM/GBSA method. As presented in Table 2, the highest binding free energy was observed for the c-Myc G4/EKB complex at –42.86 kcal/mol, followed by Bcl-2 G4/EKB (–31.77 kcal/mol), c-KIT G4/EKB (–29.94 kcal/mol), and K-RAS G4/EKB (–22.74 kcal/mol). Overall, van der Waals interactions (ΔE _vdw_) were the dominant stabilizing force, making the largest contribution to the total binding free energy in all the complexes. The nonpolar solvation energy (ΔG _SA_) also contributed to stabilization, albeit to a lesser extent. Conversely, the polar solvation energy (ΔG _GB_) consistently acted as the primary destabilizing factor, partially counterbalancing the stabilizing effects. These findings demonstrate that EKB has strong binding affinity for c-Myc G4 and notable affinity for Bcl-2 G4, which is consistent with the results of previous FRET experiments.

**Table:** **Table 2** Binding free energies of the four systems calculated in MM/GBSA (kcal/mol)

System	Δ *E*_vdw_	Δ *E*_ele_	ΔG _GB_	ΔG _SA_	ΔG _bind_
c-Myc G4/EKB	–61.59 ±1.86	–18.58 ± 7.09	41.93 ± 10.38	–4.62 ± 0.10	–42.86 ± 4.65
c-KIT G4/EKB	–42.98 ± 1.87	–26.74 ± 5.23	42.97 ± 8.10	–3.20 ± 0.11	–29.94 ± 4.92
Bcl-2 G4/EKB	–53.16 ± 1.26	–3.57 ± 3.05	28.06 ± 3.67	–3.10 ± 0.10	–31.77 ± 3.74
k-RAS G4/EKB	–35.24 ± 1.64	–10.73 ± 7.66	25.73 ± 6.59	–2.50 ± 0.11	–22.74 ± 4.34

**Figure 6:**
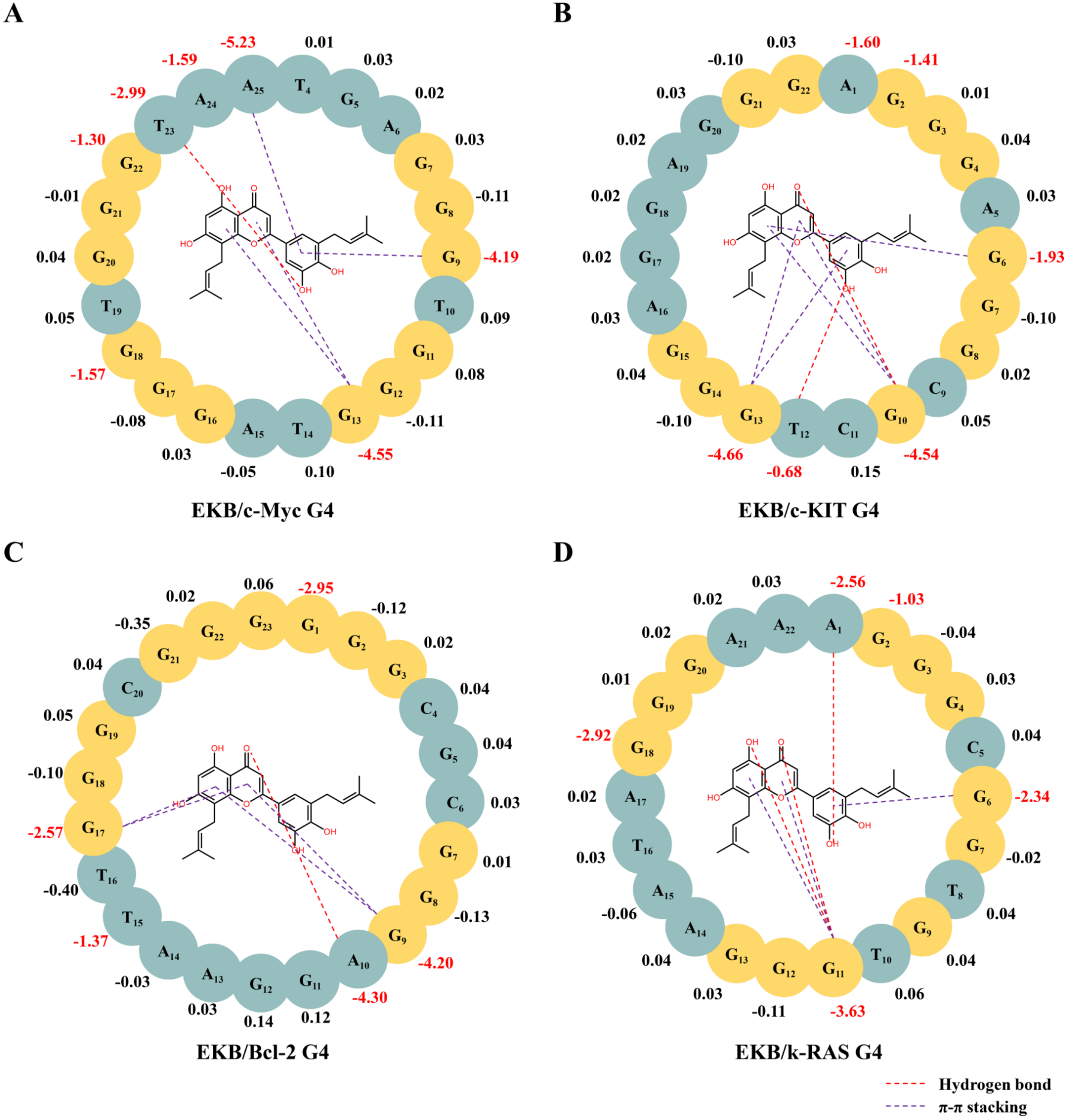
Figure 6 MM/GBSA binding free energy decomposition analysis (A–D) Decomposition of binding free energy contributions between EKB and the G4 structures of c-Myc G4 (A), c-KIT G4 (B), Bcl-2 G4 (C) and k-RAS G4 (D), with key interactions highlighted using different color annotations.

**Figure 7:**
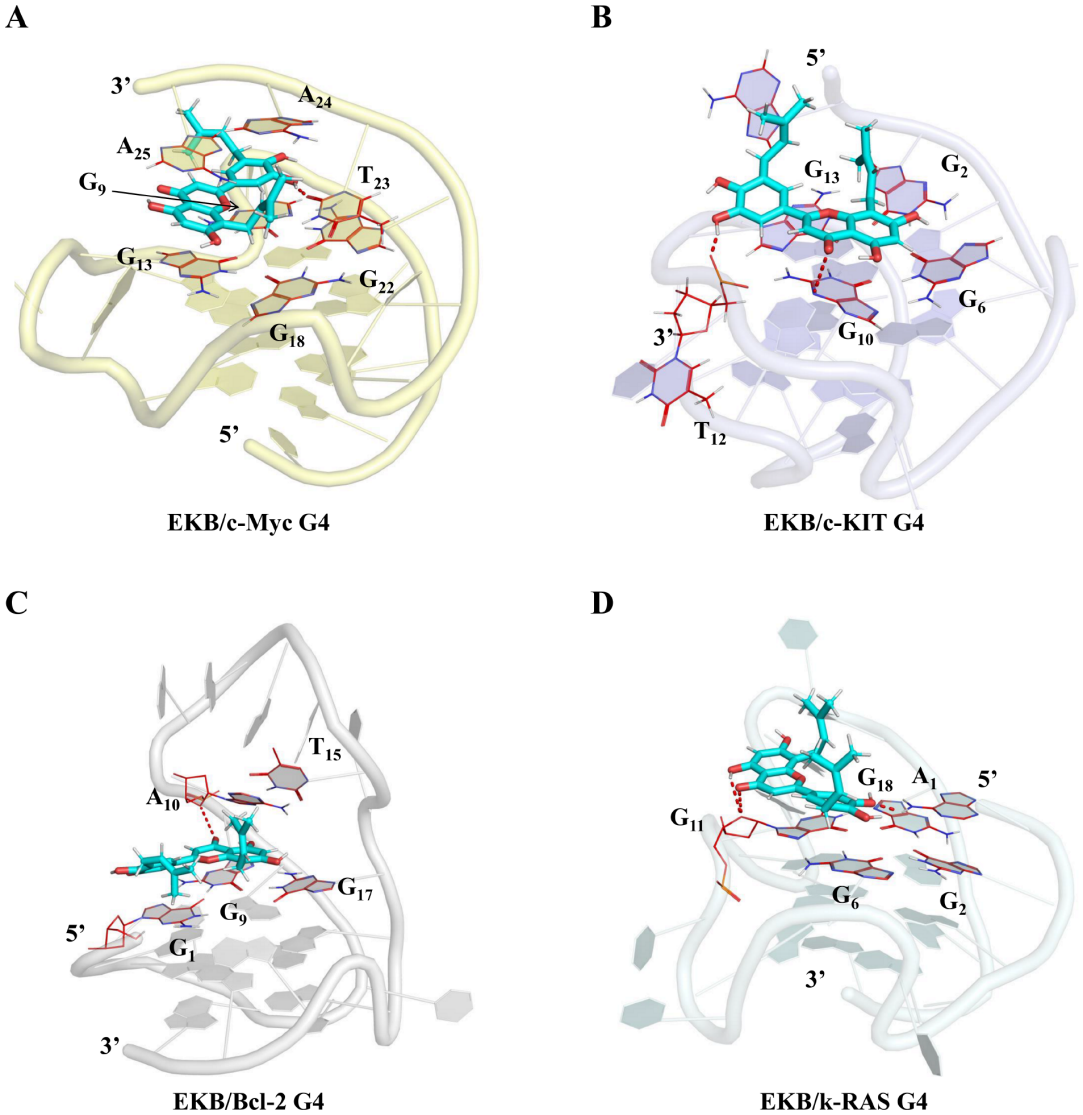
Figure 7 Visualization of the binding modes of G4/EKB complexes (A–D) Binding modes of EKB to the G4 structures of c-Myc (A), c-KIT (B), Bcl-2 (C), and k-RAS (D). The G4 structures were depicted using a cartoon representation, whereas EKB was visualized as a blue stick model. Hydrogen bond interactions are indicated by red dashed lines.

To elucidate the potential interactions between EKB and the G4 structures of c-Myc, c-KIT, Bcl-2, and k-RAS, we conducted MM/GBSA binding free energy decomposition analyses on multiple bases within these systems. This approach provided a comprehensive understanding of the contributions of individual bases in the G4 structures to their interactions with EKB. As shown in Figures 6A and 7A, EKB bound to the 3′-end of c-Myc G4 by stacking interactions. Notably, the key bases that significantly contributed to the binding energy included G9 (–4.19 kcal/mol), G13 (–4.55 kcal/mol), G18 (–1.57 kcal/mol), G22 (–1.30 kcal/mol), T23 (–2.99 kcal/mol), A24 (–1.59 kcal/mol), and A25 (–5.23 kcal/mol), with A25 exhibiting the most substantial contribution. Specifically, EKB formed π-π stacking interactions with G13 and A25 and established a hydrogen bond with T23, thereby increasing the overall binding stability.

As depicted in Figures 6B and 7B, EKB interacted with the 5′-end of the c-KIT G4 structure through stacking interactions. The key contributing bases included G6 (–1.93 kcal/mol), G10 (–4.54 kcal/mol), and G13 (–4.66 kcal/mol), with G13 and G10 contributing the most to the overall binding affinity. Additionally, EKB formed two hydrogen bonds with G10 and T12, further enhancing the binding stability. As shown in Figures 6C and 7C, EKB exhibited strong affinity for the 5′-end of the Bcl-2 G4 structure, with key contributions from G1 (–2.95 kcal/mol), G9 (–4.20 kcal/mol), A10 (–4.30 kcal/mol), T15 (–1.37 kcal/mol), and G17 (–2.57 kcal/mol). Notably, G9 participated in π-π stacking interactions with EKB, whereas A10 engaged in hydrogen bonding. As illustrated in Figures 6D and 7D, EKB was also able to bind to the 5′-end of k-RAS G4 by embedding itself within the G4 stacking region. The planar G-quartet composed of guanines G2 (–1.03 kcal/mol), G6 (–2.34 kcal/mol), G11 (–3.63 kcal/mol), and G18 (–2.92 kcal/mol) provided significant binding affinity for the interaction between EKB and the k-RAS G4 structure through its unique stacking arrangement. The localized binding mode indicated that non-covalent interactions, including π-π stacking and hydrogen bonding, worked synergistically to stabilize the G4 structure. This detailed analysis underscores the critical roles of specific nucleotides and non-covalent interactions in mediating the binding stability of EKB with G4 structures, offering valuable insights into its potential as a G4-targeting agent.

### MD simulation of G4 single-point mutants

To validate the MD simulation predictions and further investigate the sequence specificity of EKB binding, single-point G→A mutations [14] were introduced at guanine residues that contributed most significantly to the binding free energy in each G4/EKB complex. From a conformational stability perspective, these mutant complexes displayed delayed equilibration and increased RMSD values, suggesting reduced structural stability and slower dynamic convergence than the wild-type complexes did. Additionally, RMSF analysis during the final 100 ns revealed elevated nucleotide fluctuations in the mutants, further indicating reduced structural stability upon mutation ( Supplementary Figure S3).

Binding free energies were recalculated via the MM/GBSA method ( Table 3). Compared with the corresponding wild-type complexes, all the mutant complexes presented varying degrees of decreased binding affinity, highlighting the importance of these guanine residues in maintaining stable interactions with EKB. Among them, the Bcl-2 G4 (G9A) mutant displayed the most dramatic reduction, with the binding energy decreasing from –31.77 to –11.52 kcal/mol, suggesting a key role for this site in ligand recognition. The c-Myc G4 (G13A) mutant also presented a significant decrease (–35.01 kcal/mol), whereas the c-KIT G4 (G10A) mutant presented a substantial reduction to –14.76 kcal/mol. The k-RAS G4 (G11A) mutant demonstrated a moderate decrease, with a binding free energy of –18.94 kcal/mol.

**Table:** **Table 3** Binding free energy components of the mutant G4/EKB complexes calculated by MM/GBSA (kcal/mol)

System	*ΔE*_vdw_	*ΔE*_ele_	*ΔG*_GB_	*ΔG*_SA_	*ΔG*_bind_
c-Myc G4 (G13A)/EKB	–51.71 ± 1.91	–25.19 ± 8.76	46.08 ± 8.29	–4.18 ± 0.09	–35.01 ± 4.22
c-KIT G4 (G10A)/EKB	–22.95 ± 2.35	–12.50 ± 13.21	22.51 ± 11.52	–1.82 ± 0.17	–14.76 ± 4.21
Bcl-2 G4 (G9A)/EKB	–16.45 ± 1.65	–32.63 ± 6.93	39.44 ± 6.74	–1.89 ± 0.10	–11.52 ± 3.71
k-RAS G4 (G11A)/EKB	–30.55 ± 1.65	–2.02 ± 15.69	15.62 ± 14.23	–1.99 ± 0.14	–18.94 ± 4.16

Collectively, these results indicate that EKB binding is highly dependent on specific guanine residues. Disruption of these critical bases significantly weakens binding stability, further supporting a sequence- and structure-selective G4 recognition mechanism.

## Discussion

Since the initial report by Hurley *et al*. in 2002 identifying a G4 structure in the promoter region of *c-Myc*, numerous studies have revealed that G4s form within the proximal promoters of multiple oncogenes, where they function as transcriptional regulatory elements [15]. The *c-Myc* promoter G4 remains the most well-characterized example and is known to suppress gene transcription by impeding transcription factor binding and RNA polymerase progression. Subsequently, G4 elements have been identified in the promoters of *c-MYB*, *c-KIT*, *HIF1A*, *h-RAS*, *k-RAS*, and other oncogenes, underscoring the widespread role of G4s in transcriptional regulation. In addition to their role in transcription regulation, G4s play critical roles in telomere maintenance. DNA G4s are known to inhibit telomerase-mediated telomere elongation, whereas RNA G4s regulate telomere integrity through specific interactions with the telomeric protein TRF2. Additionally, G4 structures can act as replication fork barriers, induce DNA damage, and are associated with gene amplification and genomic instability in tumors [33]. Collectively, these findings highlight G4s as key participants in multiple aspects of tumorigenesis and as attractive therapeutic targets.

Over the past two decades, the development of G4-targeting small molecules has become a prominent direction in anticancer drug discovery [16]. These ligands stabilize intracellular G4s to suppress oncogene expression, interfere with telomerase activity, and activate DNA damage responses [15]. Many of these compounds can act on multiple G4 structures, including G4 clusters within single promoters, enabling the coordinated downregulation of several oncogenic drivers—a promising “multitarget” strategy to overcome tumor heterogeneity and improve therapeutic efficacy [16]. Traditionally, ideal G4 ligands are thought to be large, planar, and symmetric aromatic compounds that maximize stacking on external G-quartets [34]. However, studies by Chen *et al*. [35] have demonstrated that small, asymmetric molecules with specific functional groups may offer greater selectivity. These ligands engage in “base recruitment” by stacking on outer G-quartets while simultaneously forming hydrogen bonds and groove or loop interactions, enabling specific and stable recognition of biologically relevant G4s. To date, two G4-targeting compounds, CX-3543 and CX-5461, have entered clinical evaluation. Notably, CX-3543, which was originally designed to target RNA G4s, has completed phase I and II trials without severe G4-related toxicity, supporting the clinical viability of this approach [ 36, 37].

In the present study, we report for the first time that Epimedokoreanin B, a natural isoprenylated flavonoid, functions as a potent G4 stabilizer with significant cytotoxicity against MM cells. Compared with the known G4 ligand CX-5461, EKB exhibited lower IC _50_ values in both the U266 and RPMI-8226 cell lines, indicating superior anticancer potency. Apoptosis assays further confirmed dose-dependent cell death, supporting the potential of EKB as a G4-targeting therapeutic agent. Using BG4 immunofluorescence staining, we observed substantial G4 accumulation in EKB-treated cells, confirming its G4-stabilizing activity at the cellular level. FRET and UV-Vis titration assays demonstrated that EKB selectively stabilized G4s from the promoters of c-Myc, Bcl-2, c-KIT, and k-RAS, with no significant binding to dsDNA. EKB significantly increased the thermal stability of these G4s. Consistent with these findings, q-PCR and western blot analyses revealed pronounced suppression of oncogene expression at both the transcriptional and translational levels following EKB treatment, supporting the hypothesis that EKB exerts its antitumor effects via G4 stabilization. To functionally validate the impact on transcription, we employed a luciferase reporter assay driven by the wild-type *c-Myc* promoter. EKB treatment led to a concentration-dependent reduction in luciferase activity, indicating that EKB inhibits *c-Myc* transcription by stabilizing the G4 structure within the NHE III1 region.

MD simulations and MM/GBSA binding free energy decomposition further elucidated the specific binding modes of EKB with various G4 structures. EKB engages in π-π stacking on the G-quartet, forms hydrogen bonds with nearby residues, and interacts according to the topological features of each G4. Point mutation analyses confirmed that substitution of critical guanine residues significantly impaired binding affinity, emphasizing the importance of sequence specificity in G4-ligand recognition.

However, this study did not explore potential synergistic effects between EKB and existing clinical MM therapies. More importantly, although we demonstrated that EKB stabilizes the promoter G4s of c-Myc, Bcl-2, c-KIT, and k-RAS, the downstream signaling pathways involved remain to be fully elucidated. In conclusion, this study identified EKB as a promising multitarget G4 stabilizer with potential applications in MM therapy. Our integrative experimental and computational approach not only confirms the druggability of G4 structures but also supports the use of natural products as valuable scaffolds in G4-targeted anticancer drug development.

## Supporting information

25108Supplementary_data_revised

## Supplementary Data

Supplementary data are available at *Acta Biochimica et Biophysica Sinica* online.
