# Supplementary material for: Directly targeting G-quadruplexes contributes to the anti-multiple myeloma efficacy of Epimedokoreanin B: Identification of EKB as a potential G4 stabilizer
Source: Acta Biochim Biophys Sin (Shanghai). 2025 Aug 14;58(4):819–32. doi: 10.3724/abbs.2025110 (PMC13107025; doi:10.3724/abbs.2025110)
Supplement: 25108Supplementary_data_revised [file 25108Supplementary_data_revised.docx]

**Supplementary Table S1.** **The specific sequences used in this study**

| Oligonucleotide | Sequence (5′→3′) |
| --- | --- |
| dsDNA | CAATCGGATCGAATTCGATCCGATTG |
| Telomeric G4 | GGGGAGGGGCTGGGAGGGCCCGGAGGGGGCTGGGCCGGGGACCCGGGAGGGGTCGGGACGGGGCGGGG |
| c-Myc G4 | GCGCTTATGGGGAGGGTGGGGAGGGTGGGGAAGGTGGGGAGGAGAC |
| c-KIT G4 | AGGGAGGGCGCTGGGAGGAGGGG |
| Bcl-2 G4 | CGGGCGGGAGCGCGGCGGGCGGGCGGG |
| k-RAS G4 | AGGGCGGTGTGGGAAGAGGGAAGAGGGGGAGG |

**Supplementary Table S2.** **Melting temperature of EKB in response to diverse target oligonucleotides**

| Tm/ºC | dsDNA | Telomeric | c-Myc | c-KIT | Bcl-2 | k-RAS |
| --- | --- | --- | --- | --- | --- | --- |
| Tm (G4) | 85.24 | 61.44 | 61.76 | 78.33 | 45.65 | 55.33 |
| Tm (1 eq) | 83.37 | 62.12 | 62.54 | 81.89 | 50.06 | 56.44 |
| Tm (2 eq) | 84.84 | 61.56 | 62.87 | 81.44 | 50.04 | 56.33 |
| Tm (4 eq) | 82.94 | 62.59 | 63.57 | 82.02 | 51.18 | 57.01 |
| Tm (8 eq) | 84.08 | 63.56 | 65.66 | 82.5 | 52.74 | 58.55 |


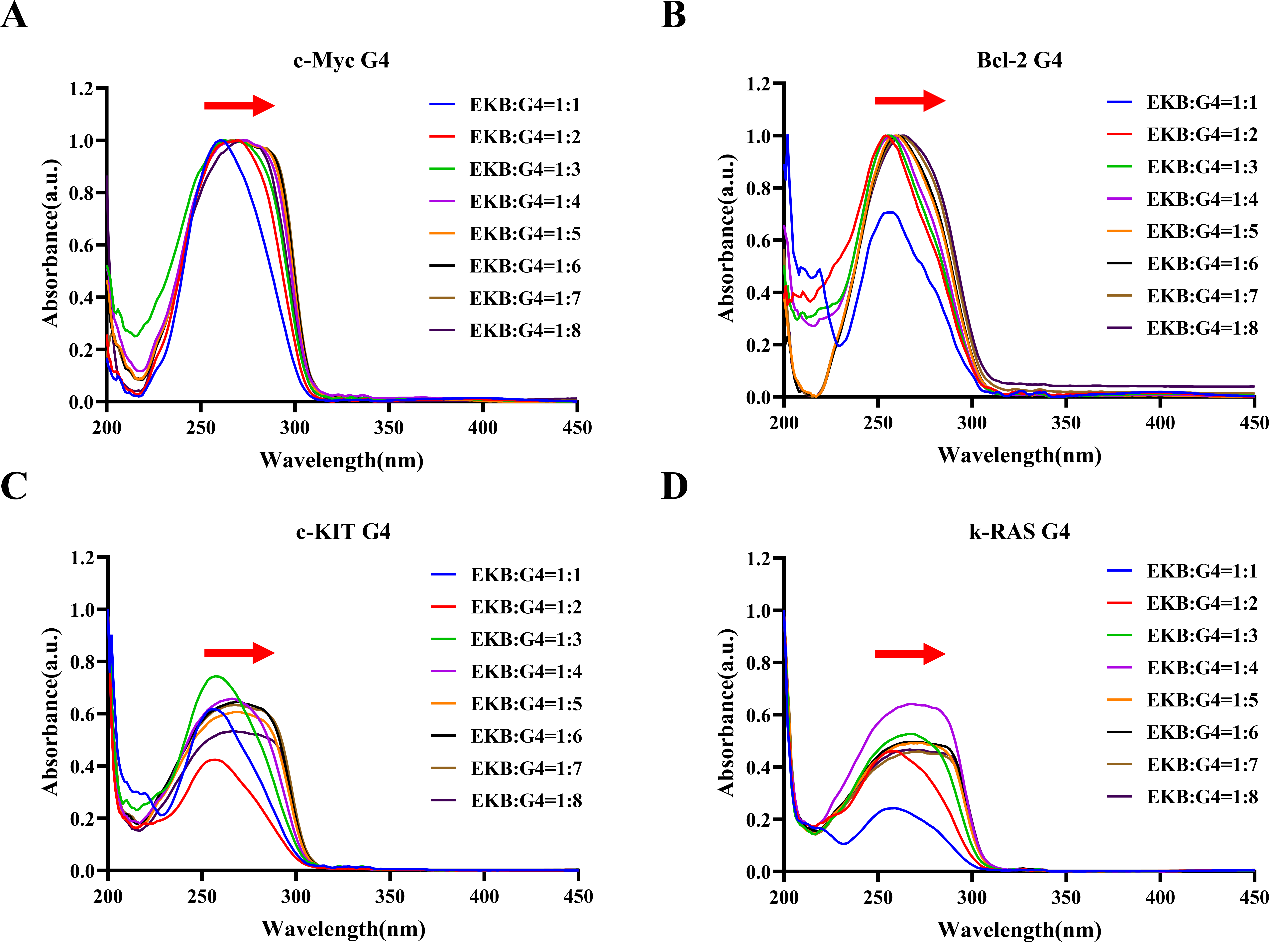


**Supplementary Figure S1. UV-visible absorption spectra of G4 DNAs titrated with increasing concentrations of EKB** UV-visible spectra of G4 DNA sequences derived from the promoter regions of (A) c-Myc, (B) Bcl-2, (C) c-KIT, and (D) k-RAS. Spectra were collected in 10 mM Tris-HCl (pH 7.4) containing 100 mM KCl at room temperature.


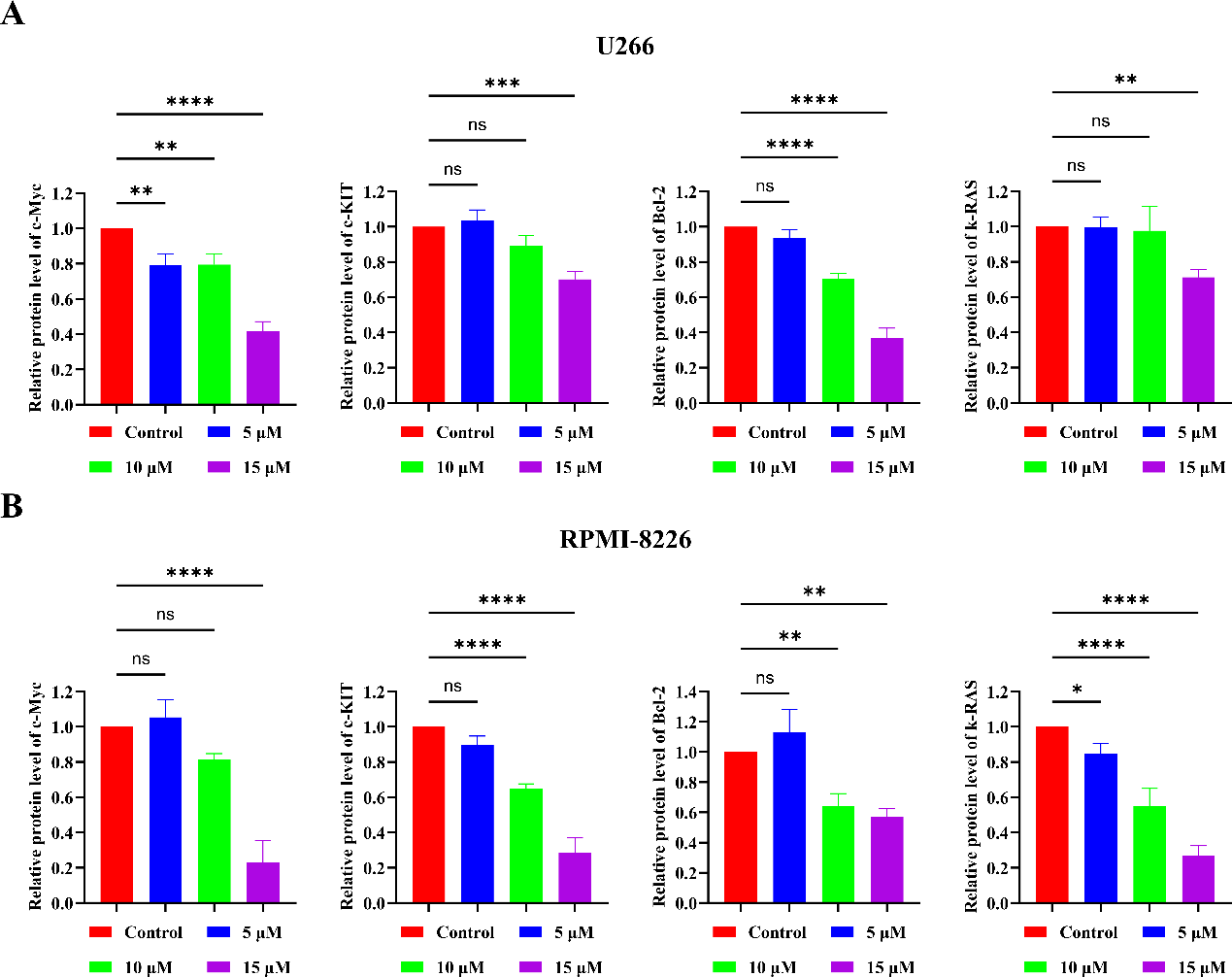


**Supplementary Figure S2. Relative protein levels of c-Myc, c-KIT, Bcl-2, and k-RAS** These proteins were measured by western blot analysis in U266 (A) and RPMI-8226 (B) cells following treatment with different concentrations of EKB (5, 10, and 15 μM) for 24 h. GAPDH was used as the internal control for normalization. Data are shown as the mean ± SD (*n* = 3). Statistical significance was assessed by one-way ANOVA with Tukey’s test (ns, not significant, ***P* < 0.01, *****P* < 0.0001).


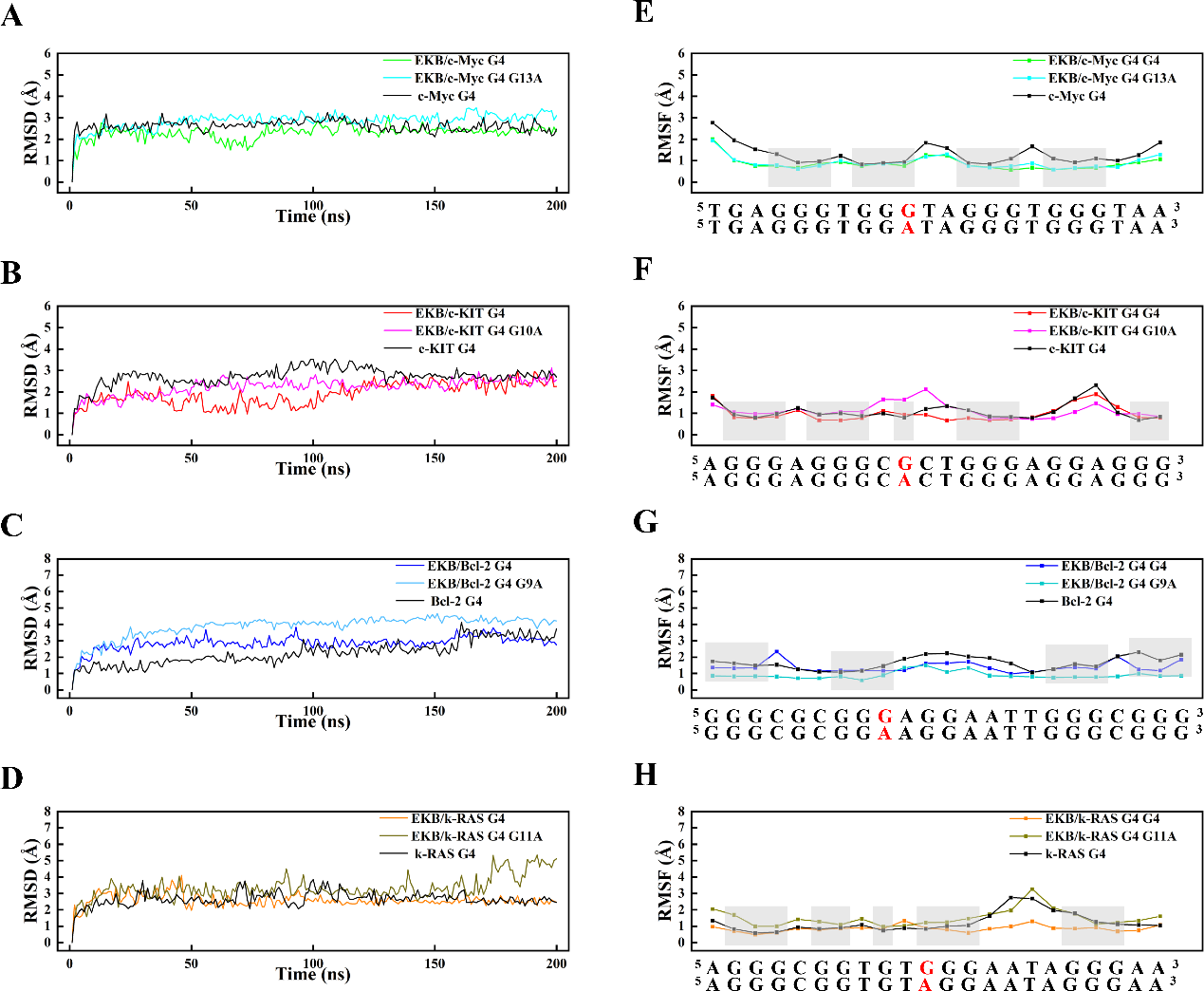


**Supplementary Figure S3.** **Structural stability analysis of wild-type and mutant G4/EKB complexes** (A−D) (RMSD plots of the c-Myc G4 (A), c-KIT G4 (B), Bcl-2 G4 (C), and k-RAS G4 (D) complexes with EKB and their corresponding single-point mutants (G→A) over 200 ns molecular dynamics simulations. (E−H) RMSF profiles of each nucleotide in the wild-type and mutant G4 structures of c-Myc (E), c-KIT (F), Bcl-2 (G), and k-RAS (H), in complex with EKB. Mutated residues are highlighted in red within the sequences.
